# Involved-field radiotherapy (IFRT) versus elective nodal irradiation (ENI) in combination with concurrent chemotherapy for 239 esophageal cancers: a single institutional retrospective study

**DOI:** 10.1186/s13014-015-0482-9

**Published:** 2015-08-14

**Authors:** Hideomi Yamashita, Ryousuke Takenaka, Mami Omori, Toshikazu Imae, Kae Okuma, Kuni Ohtomo, Keiichi Nakagawa

**Affiliations:** Department of Radiology, University of Tokyo Hospital, 7-3-1, Hongo, Bunkyo-ku, Tokyo, 113-8655 Japan

## Abstract

**Background:**

This retrospective study on early and locally advanced esophageal cancer was conducted to evaluate locoregional failure and its impact on survival by comparing involved field radiotherapy (IFRT) with elective nodal irradiation (ENI) in combination with concurrent chemotherapy.

**Methods:**

We assessed all patients with esophageal cancer of stages I-IV treated with definitive radiotherapy from June 2000 to March 2014. Between 2000 and 2011, ENI was used for all cases excluding high age cases. After Feb 2011, a prospective study about IFRT was started, and therefore IFRT was used since then for all cases. Concurrent chemotherapy regimen was nedaplatin (80 mg/m^2^ at D1 and D29) and 5-fluorouracil (800 mg/m^2^ at D1-4 and D29-32).

**Results:**

Of the 239 consecutive patients assessed (120 ENI vs. 119 IFRT), 59 patients (24.7 %) had stage IV disease and all patients received at least one cycle of chemotherapy. The median follow-up time for survivors was 34.0 months. There were differences in 3-year local control (44.8 % vs. 55.5 %, *p* = 0.039), distant control (53.8 % vs. 69.9 %, *p* = 0.021) and overall survival (34.8 % vs. 51.6 %, *p* = 0.087) rates between ENI vs. IFRT, respectively. Patients treated with IFRT (8 %) demonstrated a significantly lower risk (*p* = 0.047) of high grade late toxicities than with ENI (16 %). IFRT did not increase the risk of initially uninvolved or isolated nodal failures (27.5 % in ENI and 13.4 % in IFRT).

**Conclusions:**

Nodal failure rates in clinically uninvolved nodal stations were not increased with IFRT when compared to ENI. IFRT also resulted in significantly decreased esophageal toxicity, suggesting that IFRT may allow for integration of concurrent systemic chemotherapy in a greater proportion of patients. Both tendencies of improved loco-regional progression-free survival and a significant increased overall survival rate favored the IFRT arm over the ENI arm in this study.

## Introduction

Concurrent chemoradiotherapy (CCRT) is well established as a standard approach to treat locally advanced esophageal cancer [1–3]. It has shown a 2-year local control rate of 55 % and a 5-year survival rate of 25 %, accompanied with severe therapy-induced side effects. Herskovic *et al.* [1] reported that the rates of severe and life-threatening side effects were 44 and 20 %, respectively. A 2 % death rate was observed to be iatrogenic as well.

Based on the results of RTOG 85–01 [1], CCRT has been broadly applied as a standard management for patients with inoperable esophageal cancer. In that study, 30 Gy was given to the whole esophagus followed by a cone down of 20 Gy to the primary tumor with 5-cm proximal and distal margins. However, the loco-regional failure and life-threatening side effects were as high as 50 and 20 %, respectively. Therefore, the RTOG 94–05 trial was conducted [2] in which 50.4 Gy was administered to the radiation field with superior and inferior borders of 5 cm beyond the primary tumor, and followed by a cone down of 14.4 Gy to the primary tumor with 2-cm proximal and distal margins. Unfortunately, no apparent benefit was obtained and the treatment related deaths were even higher. It is conceivable that the superior and inferior borders of 5 cm beyond the primary tumor did not cover high-risk sub-clinical metastatic areas. Recently, Zhao *et al.* [4] evaluated the appropriate target volumes in radiotherapy-alone of 68.4 Gy in 41 fractions using late-course accelerated hyperfractionated three-dimensional conformal radiotherapy (3D-CRT) for esophageal squamous cell carcinoma (SqCC). They concluded that the omission of elective nodal irradiation (ENI) was not associated with a significant amount of failure in lymph node (LN) regions not included in the planning target volume (PTV).

Since the early 1980s, Japanese surgeons have practiced 3-field regional LN dissections for esophageal cancer [5, 6]. There are some reports indicating that prophylactic 3-field LN dissections for esophageal cancer can lead to an improved survival [7, 8]. In accordance with the concept of 3-field LN dissections in curative surgery, ENI had been adopted for definitive CCRT at our institution until 2011 [9], inasmuch as the benefit of ENI in CCRT for thoracic esophageal cancer lacked consensus [10–14].

There is also a lack of consensus on the design of an optimal radiation field. Recently, several different investigators reported conflicting results on the role of extensive or elective nodal irradiation in definitive CRT for esophageal cancer [2, 4, 9, 15, 16].

The trend towards treating locally advanced esophageal cancer with IFRT has generated concern for the increased risk of nodal failure in untreated nodal stations, as clinically uninvolved lymph nodes may harbor microscopic disease. Therefore, by not including clinically uninvolved mediastinal lymph nodes in the radiation field, IFRT may potentially result in progression of subclinical nodal disease.

The purpose of this study was to evaluate the clinical outcomes and toxicities in patients with esophageal cancer treated definitively with either ENI or IFRT combined with chemotherapy.

## Materials and methods

This is a retrospective review of 239 consecutively treated patients diagnosed with inoperable esophageal cancer of stages I-IV who were treated with definitive intent at the University of Tokyo Hospital from June 2000 to March 2014. This study has been done retrospectively and was approved by a local ethic/IRB board (No. 3372). These dates were chosen to minimize the impact of differences in treatment delivery excluding the change of radiation field concept halfway in the study period and stage migration, as 3D-conformal treatment planning and formal staging with ^18^FDG-PET were initiated at the University of Tokyo Hospital in 2006. This study was approved by the University of Tokyo Hospital Institutional Review Board. Between 2000 and 2011, ENI was used for all cases excluding high age cases and IFRT was used for high age cases from this time. After Feb 2011, we started a prospective study on IFRT, and since then IFRT has been used for all cases. In our institution, the patients who underwent non-surgical therapy were either technically unresectable, refused surgery, or were medically unfit. In our institution, 462 patients have been operated during the same period.

### Radiotherapy planning in ENI arm

**Fig. 1:**
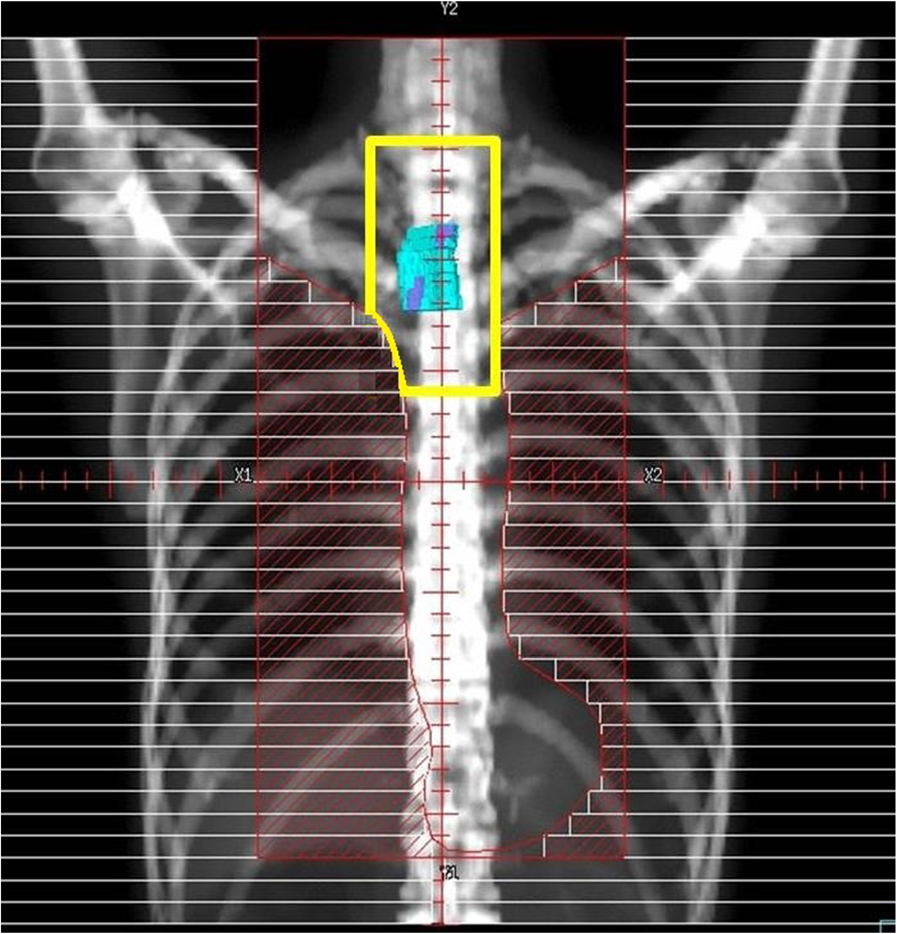
Beam’s eye view of both ENI and IFRT for upper thoracic esophageal cancer (sky blue = gross tumor, red = ENI, and yellow = IFRT)

Patients in the ENI arm were treated with 50–50.4 Gy delivered in 1.8–2 Gy per fraction over 5–5.6 weeks. Gross Tumor Volume (GTV) was defined for each subject as tumor volume was visualized on CT and endoscopic extension. All LNs with a diameter at least one cm in short axis in CT or positive by FDG-PET (excluding physiological accumulation) were included in the GTV. To sum up, GTV included primary cancer and metastatic lymph nodes. Clinical target volume (CTV) was defined as the whole thoracic esophagus (= from the supraclavicular fossae to the esophagogastric junction) including GTV plus 5 mm margin. CTV comprised up to M1a LNs as well as regional LNs including positive LNs. The definition of regional LNs by AJCC 1997 is mediastinal and perigastric LN excluding celiac LN. The definition of M1a region by AJCC 1997 is cervical LNs in the upper thoracic, celiac LNs in the lower thoracic esophagus, and none in the middle thoracic. PTV was created by adding margins of 5–10 mm to the respective CTVs (Fig. 1). Irradiated dose was specified to the ICRU point. Treatment was entirely 3D-planned, and dose homogeneity criteria within respective PTVs had to be within 95–107 % of the prescribed dose even if the field-in-field technique was used. At least four fields were used: two anterior–posterior opposed fields, and two anterior–posterior oblique opposed fields to remove the spinal cord from the radiation fields. Also, one or two beams were added with the field-in-field technique if necessary. Mean lung dose had to be kept at or below 20 Gy and V20 (= the lung volume rate receiving over 20 Gy) < 20 %. Spinal cord dose had to be kept at or below 45 Gy. Treatment was delivered by linear accelerators with 6–10 MV photons.

### Radiotherapy planning in IFRT arm

Patients in the IFRT arm were treated with 50.4 Gy delivered over 5.6 weeks at 1.8 Gy per fraction. Tumor volume was visualized on computed tomography (CT) and/or PET and endoscopic extension and used to define gross tumor volume (GTV) for each patient. All LNs with a diameter at least one cm in short axis in CT or positive by ^18^FDG-PET (excluding physiological accumulation) were included in the GTV. The clinical target volume (CTV) was generated by using no radial margin and 2 cm longitudinal margins to the GTV-primary, and by using no margin for the GTV-LNs. The planning target volume (PTV) was then generated by applying a 5 mm radial margin and a 10 mm longitudinal margin to the CTV (Fig. 1). The mediastinal LNs were not electively irradiated in this study. PTV-min was more than 90 % of prescribed dose and PTV ≥ 107 % was less than 5 %.

At least four fields were used: two anterior–posterior opposed fields, and two anterior–posterior oblique opposed fields to remove the spinal cord from the radiation fields and if necessary, one/two beams were added with the field-in-field technique. The dose constraints for OARs were similar to those of ENI arm plus the constraint for the heart was D75 % < 45 Gy and mean heart dose < 30 Gy.

A positive indication for the treatment field was signified when the ^18^FDG-PET standardized uptake value on the highest image pixel in the tumor regions (SUVmax) was 2.5 or more. A PET-CT match was not performed for delineation for GTV. Image guided RT was performed using cone beam CT each day.

### Chemotherapy regimen

All patients received chemotherapy concurrently with irradiation. Chemotherapy consisted of two cycles of 5-fluorouracil (5-FU) (800 mg/m^2^/day, days 1–4 & days 29–32, continuous) combined with cisplatin or nedaplatin (NDP) (80 mg/m^2^, day 1 & day 29, bolus); standard techniques were used for hydration and alkalization. For a case 75 years or older, reductions were made to an 80 % dose. Chemotherapy was started on the first day of irradiation. After concurrent CRT, in the adjuvant setting, an additional one or two cycles of the same dose of chemotherapy were given for patients who still had sufficient bone-marrow function and performance status and who did not refuse additional chemotherapy.

### Follow-up and evaluation criteria

After completion of treatment, patients were reviewed within 4–6 weeks, then every 3 months in the first 2 years, every 4 months in the third year, and every 6 months thereafter. Physical examinations included serum tumor markers of carcino-embryonic antigen (CEA), squamous cell carcinoma-related antigen (SCC), cytokeratin 19 fragment (CYFRA), and p53 (each month) [17–20]. Other studies performed routinely were upper gastrointestinal endoscopy (+/− biopsy) every 3–4 months, CT scans of the thorax and upper abdomen every 2–3 months, and PET/CT scans every 6–12 months or when a recurrence was suspected by other examinations.

### Response and toxicity criteria

After completion of concurrent CRT, tumor response was evaluated with thoracic CT scans in accordance with Response Evaluation Criteria in Solid Tumors Group 1.0 (RECIST1.0). An elective nodal failure (ENF) was defined as a nodal failure in the elective irradiation region in the ENI arm and an uninvolved nodal failure out of the irradiation field in the IFRT arm. Involved-field nodal failure (IFNF) was defined as a failure in the nodal region with nodal metastasis before CRT. Loco-regional failure included primary tumor failure, ENF and IFNF. Local progression-free survival (LPFS) was recorded from the beginning of induction chemotherapy to the time of primary tumor failure, ENF or IFNF. During radiotherapy, acute radiation-induced pneumonitis and esophagitis as well as changes in body weight of each patient were recorded, and a complete blood count was performed at least once a week. Acute hematologic toxicities and weight loss were classified in accordance with the National Cancer Institute Common Toxicity Criteria (CTCAE) version 4.0. Acute and late toxicities of lung and esophagus were evaluated according to RTOG criteria [21].

### Statistical analysis

Comparisons of patient and tumor characteristics, toxicity, and site of first failure were performed with *χ*2 tests, 2-sample *t*-tests or Wilcoxon Rank Sum tests.

The Kaplan-Meier method was used to estimate survival data. The distribution of survival time between arms was tested by the log-rank method. Student’s *t*-test was used for comparison of means. Fisher’s exact test was used for comparisons of categorical data. The multivariate analysis was performed by the proportional hazard model to address such confounding factors as clinical stage, TNM stage, age, location of primary tumor, and the number of chemotherapy cycles with RT field of ENI versus IFRT on OS and PFS. All *p* values were based on a 2-sided test, and the differences were regarded as statistically significant when *p* < 0.05.

## Results

### Patient characteristics

There were 239 consecutive patients with esophageal cancer enrolled in this study. The characteristics of the 239 eligible patients are shown in Table 1.

**Table 1:** Patient and tumor characteristics

	ENI		IFRT		
Factor	N	%	N	%	*P* value
Total	120		119		
Sex					
Female	13	11 %	21	18 %	0.13
Male	107	89 %	98	82 %	
Age (y.o.)					
Range	46–83		44–86		0.11
Median	67		68		
Location					
Ce	4	3 %	6	5 %	0.63
Ut	19	16 %	20	17 %	
Mt	56	47 %	61	51 %	
Lt	41	34 %	32	27 %	
K-PS					
-80 %	41	34 %	30	25 %	0.13
90–100 %	79	66 %	89	75 %	
Cycle number of CTx					
1	28	23 %	15	13 %	0.062
2	49	41 %	44	37 %	
3	13	11 %	15	13 %	
4	30	25 %	45	38 %	
1–2	77	64 %	59	50 %	0.023
3–4	43	36 %	60	50 %	
FDG-PET scan					
Conducted No. SUV-max in primary tumor	61	51 %	103	87 %	<0.0001
0–5.0	17	28 %	25	21 %	0.59
5.0–10.0	14	23 %	19	16 %	
10.0-	30	49 %	59	50 %	
Clinical T classification					
T1	27	23 %	13	11 %	0.0004
T2	19	16 %	14	12 %	
T3	51	43 %	40	34 %	
T4	23	19 %	52	44 %	
Clinical N classification					
N0	46	39 %	39	33 %	0.37
N1	74	62 %	80	67 %	
Clinical M classification					
M0	80	67 %	97	82 %	0.0040
M1	40	34 %	20	17 %	
Clinical stage					
I	21	18 %	21	18 %	0.24
II	28	24 %	19	16 %	
III	32	27 %	45	38 %	
IV	39	33 %	34	29 %	
Histopathological type					
Squamous cell carcinoma	113	95 %	109	92 %	0.44
Others	7	6 %	10	8 %	
CTx regimen					
CDDP/5-FU	40	34 %		%	
NDP/5-FU	80	67 %		%	
NDP/TS-1	0	0 %		%	
Irradiated total dose					
50Gy/50.4Gy	108	91 %	118	99 %	0.85
Under 50 Gy	3	3 %	1	1 %	
Over 50.4 Gy	9	8 %	0	0 %	

Abbreviation: *ENI* Elective nodal irradiation, *IFRT* Involved-field radiotherapy, *Ce* Cervical esophagus, *Ut* Upper thoracic esophagus, *Mt* Middle thoracic esophagus, *Lt* Lower thoracic esophagus, *K-PS* Karnofsky performance status, *CTx* Chemotherapy, *FDG-PET* Fluorodeoxyglucose positron emissioin tomography, *SUV* Standardized uptake value, *N* Number, *No*. Number

### Treatment results

Only one cycle of chemotherapy was performed in 43 patients (28 in ENI and 15 in IFRT). Four patients (3 in ENI and 1 in IFRT) withdrew from the radiation plan. The RT plan in the IFRT and ENI arms was completed by 117 and 118 patients, respectively.

### Locoregional failure, distant metastasis, and survival

For the first recurrent site, 41 (34 %) patients in the ENI arm and 30 (25 %) patients in the IFRT arm experienced loco-regional failure (i.e. ENF, IFNF, or primary recurrence) (*p* = 0.13) (Table 2). Among them, 30 (25 %) and 23 (19 %) patients encountered primary tumor failure alone in the ENI and IFRT arms, respectively. For the first recurrent site in the IFRT arm, isolated-ENF and isolated-IFNF was present in 2 and 7 patients, respectively. In the ENI arm, 3 patients experienced isolated-ENF. For the first recurrent site, distant metastases were seen in 48 (40 %) patients in the ENI arm and 25 (21 %) patients in IFRT arm (Table 2).

**Table 2:** Results between ENI and IFRT

	ENI		IFRT		*p* value
	N	%	N	%	
State at analysis					
Alive	20	17 %	71	60 %	<0.0001
Dead	100	84 %	48	40 %	
Follow-up time of all cases					
Median	18 mo				
Range	1–169 mo				
Follow-up time of alive cases					
Median	34 mo				
Range	2–154 mo				
Residual (= non-CR)	20	17 %	6	5 %	0.0039
First locoregional REC	41	34 %	30	25 %	0.13
Primary	30		23		
Lymph node	20		9		
(Elective nodal region)	(4)		(2)		
Both	9		2		
First distant REC	48	40 %	25	21 %	0.0014
Lymph node	13		7		
Lung	20		8		
Bone	6		3		
Liver	6		5		
Salvage surgery after REC	11	9 %	3	3 %	0.029
Grade 3–5 non-hematological toxicities by RTOG	19	16 %	9	8 %	0.047
Lung	7		3		
Heart	5		0		
Esophagus	2		2		

Abbreviation: *ENI* Elective nodal irradiation, *IFRT* Involved-field radiotherapy, *CR* Complete response, *REC* Recurrence, *RTOG* Radiation Therapy Oncology Group, *N* Number

**Fig. 2:**
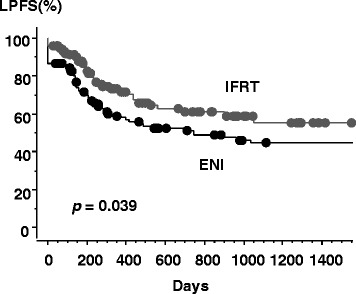
Local progression-free survival curves for patients with IFRT or ENI. Gray line = IFRT arm, black line = ENI arm, circle = censored value

Ninety-one patients remained alive at the time of analysis, with a median follow-up of 34.0 months in survivors (2.0 – 154 months). The median LPFS time was not available in the IFRT arm. The 1-, 2-, and 3-year LPFS rates were 58.9, 51.3, and 44.8 %, respectively, in the ENI arm, versus 73.0, 61.0, and 55.5 % in the IFRT arm (*p* = 0.039 by log-rank test) as shown in Fig. 2. The 1-, 2-, and 3-year distant metastasis-free survival rates were 66.1, 56.5, and 53.8 %, respectively, in the ENI arm, versus 82.4, 69.9, and 69.9 % in the IFRT arm (*p* = 0.021).

**Fig. 3:**
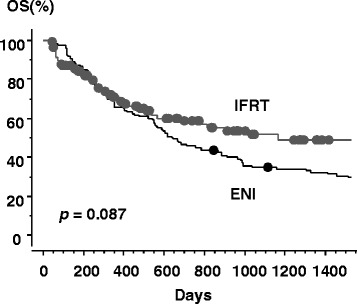
Overall survival curves for patients with IFRT or ENI. Gray line = IFRT arm, black line = ENI arm, circle = censored value

**Fig. 4:**
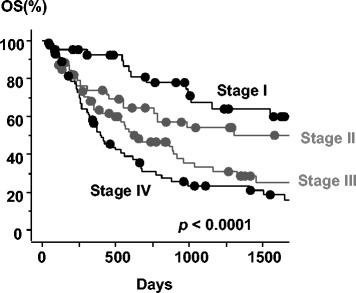
Overall survival curves for patients with stages I, II, III, and IV

The median survival time (MST) was 21.3 months [95 % confidence interval (CI), 16.1–26.5 months] in the ENI arm versus 38.9 months in the IFRT arm (95 % CI, 14.9–62.8 months). The 1-, 2-, and 3-year overall survival (OS) rates were 65.8, 45.8, and 34.8 %, respectively, in the ENI arm, versus 70.8, 58.7, and 51.6 % in the IFRT arm (Fig. 3). There was no statistical difference in overall survival between the two arms (*p* = 0.087). The 1-, 2-, and 3-year OS rates were 92.5, 78.0, and 67.6 % in stage I, 73.9, 64.5, and 53.9 % in stage II, 63.2, 46.6, and 33.3 % in stage III, and 54.8, 30.6, and 23.5 % in stage IV (*p* < 0.0001), respectively, as shown in Fig. 4 and Table 3. Besides this, there was significantly statistical difference in OS among clinical T stages (*p* < 0.0001), between ≥ 70 and < 70 years old (*p* = 0.049), and among one, two, three, and four cycles of chemotherapy (*p* = 0.0004) (Table 3). There was no statistical difference in OS between SqCC and other histopathological types (*p* = 0.64), among primary tumor locations (*p* = 0.56), and between 50 Gy and other doses (*p* = 0.82).

**Table 3:** Overall survival by characteristics

	Overall survival rate			
Clinical stage	1-y	2-y	3-y	*p* value
I	92.5 %	78.0 %	67.6 %	
II	73.9 %	64.5 %	53.9 %	
III	63.2 %	46.6 %	33.3 %	
IV	54.8 %	30.6 %	23.5 %	
Clinical T stage				<0.0001
cT1	87.2 %	75.4 %	67.5 %	
cT2	77.7 %	67.6 %	59.1 %	
cT3	65.0 %	48.2 %	35.7 %	
cT4	52.9 %	25.0 %	15.1 %	
Age (years old)				0.049
> 70	60.5 %	44.4 %	34.6 %	
< 70	72.9 %	55.8 %	45.4 %	
Cycles of chemotherapy				0.0004
One	43.2 %	29.3 %	17.6 %	
Two	67.5 %	51.3 %	41.8 %	
Three	79.2 %	66.7 %	53.5 %	
Four	77.1 %	56.7 %	49.3 %	

All high age patients were excluded from both groups. For only 214 patients with less than 80 years old, the 1-, 2-, and 3-year OS rates were 67.6, 46.8, and 25.9 % in the ENI arm, and 71.1, 57.7, and 43.3 % in the IFRT arm (*p* = 0.16), respectively. Moreover, for only 143 patients with less than 70 years old, the 1-, 2-, and 3-year OS rates were 69.4, 47.2, and 26.1 % in the ENI arm, and 73.9, 59.9, and 43.4 % in the IFRT arm (*p* = 0.17), respectively.

To ensure that these factors did not confound the relationship between radiation treatment technique (ENI vs. IFRT) and survival, a multivariable logistic regression analysis was performed using OS and PFS as the primary outcome. Other potential predictors, such as age, clinical-stage, T-stage, N-stage, M-stage, primary tumor location, and the total number of chemotherapy cycles were analyzed. When these were included in a multivariable logistic regression model, clinical-stage, T-stage, N-stage, M-stage, and the total number of chemotherapy cycles were found to independently predict longer OS and/or PFS (Table 4).

**Table 4:** Multivariate analysis

Factors	Overall survival			Progression-free survival		
	*p* - value	Odds ratio	95 % confidence interval	*p*- value	Odds ratio	95 % confidence interval
Comparison 1						
RT field	0.31			0.15		
ENI		1.21	0.84–1.73		1.32	0.90–1.93
IFRT		1			1	
M-stage	<0.0001			0.0001		
M0		0.47	0.33–0.67		0.47	0.32–0.69
M1		1			1	
Comparison 2						
RT field	0.036			0.024		
ENI		1.48	1.03–2.13		1.55	1.06–2.26
IFRT		1			1	
Age (y.o.)	0.092			0.49		
−70		0.74	0.52–1.05		1.15	0.77–1.72
71-		1			1	
Comparison 3						
RT field	0.011			0.0009		
ENI		1.60	1.11–2.29		1.90	1.30–2.79
IFRT		1			1	
T-stage	<0.0001			<0.0001		
T1	<0.0001	0.22	0.13–0.39	<0.0001	0.11	0.053–0.23
T2	0.0008	0.38	0.21–0.67	0.0010	0.36	0.19–0.66
T3	0.0021	0.53	0.36–0.80	0.013	0.59	0.39–0.89
T4		1			1	
Comparison 4						
RT field	0.017			0.0073		
ENI		1.75	1.10–2.76		1.91	1.19–3.07
IFRT		1			1	
N-stage	<0.0001			<0.0001		
N0	<0.0001	0.21	0.10–0.43	<0.0001	0.15	0.070–0.33
N1	0.0090	0.38	0.19–0.79		0.44	0.21–0.91
N2	0.13	0.54	0.24–1.21		0.55	0.24–1.24
N3		1			1	
Comparison 5						
RT field	0.082			0.040		
ENI		1.38	0.96–1.97		1.48	1.02–2.16
IFRT		1			1	
Location	0.53			0.14		
Cervix	0.44	0.66	0.23–1.90	0.14	0.34	0.081–1.44
Upper thorax	0.15	0.69	0.43–1.14	0.097	0.66	0.41–1.08
Middle thorax	0.33	0.79	0.49–1.27	0.038	0.59	0.37–0.97
Lower thorax		1			1	
Comparison 6	0.26			0.065		
RT field		1.24	0.85–1.82		1.45	0.98–2.16
ENI		1			1	
IFRT						
CTx cycle	0.002			0.083		
1	0.0012	2.29	1.39–3.78	0.50	1.20	0.70–2.04
2	0.30	1.27	0.81–1.98	0.27	0.78	0.50–1.22
3	0.48	0.78	0.39–1.55	0.042	0.45	0.21–0.97
4		1			1	
Comparison 7						
RT field	0.099			0.017		
ENI		1.35	0.94–1.93		1.58	1.09–2.30
IFRT		1			1	
Stage	<0.0001			<0.0001		
I	<0.0001	0.30	0.17–0.53	<0.0001	0.12	0.051–0.28
II	0.0009	0.43	0.26–0.71	0.015	0.53	0.32–0.88
III	0.12	0.73	0.49–1.09	0.53	0.88	0.58–1.33
IV		1			1	
Comparison 8						
RT field	0.16			0.10		
ENI		1.30	0.90–1.88		1.38	0.94–2.03
IFRT		1			1	
Age (y.o.)	0.028	1.02	1.003–1.045		1.002	0.98–1.02
M-stage	<0.0001					
M0		0.42	0.29–0.60		0.47	0.31–0.70
M1		1			1	
Comparison 9						
RT field	0.34			0.054		
ENI		1.21	0.82–1.77		1.48	0.99–2.20
IFRT		1			1	
Stage	<0.0001			<0.0001		
I	<0.0001	0.22	0.12–0.42	<0.0001	0.094	0.033–0.27
II	0.0018	0.42	0.24–0.72	0.048	0.58	0.34–0.99
III	0.14	0.74	0.49–1.11	0.75	0.93	0.60–1.44
IV		1			1	
CTx cycle	0.0006			0.066		
1	0.0002	2.62	1.58–4.35	0.12	1.52	0.89–2.59
2	0.13	1.42	0.90–2.24	0.95	0.98	0.62–1.55
3	0.62	0.84	0.42–1.67	0.078	0.50	0.24–1.08
4		1			1	

*RT* Radiation therapy, *CTx* Chemotherapy, *ENI* Elective node irradiation, *IFRT* Involved-field radiation therapy

### Acute toxicities

In the ENI arm, during CRT, when hematological adverse events were studied in the acute phase of all 120 patients, leukopenia of grades 3 and 4 was seen in 59 (49 %) and 29 patients (24 %), anemia in 22 (18 %) and 14 (12 %) patients, and thorombocytopenia in 23 (19 %) and 20 patients (17 %), respectively. Grade 4 and grades 3–4 of acute hematological adverse events were not seen in 85 (71 %) and 28 patients (23 %), respectively. On the other hand, for non-hematological side effects, acute radiation esophagitis of grades 2, 3, and 4 was seen in 35 (29 %), 28 (23 %), and 2 patients (2 %), respectively. One patient suffered from a treatment-related death (= grade 5) by esophageal bleeding at 2.1 months after starting definitive CRT. Diarrhea of grade 3 was seen in one patient, and 5-FU induced hyper-ammonemia was seen in another patient. Severe bacterial pneumonia including sepsis was seen in 9 patients (7 %), and 3 of these patients died of the side effect (= grade 5).

In the IFRT arm, regarding worst toxicities throughout the treatment period, Grade ≥ 3 toxicities of leukopenia, anemia, and thrombocytopenia occurred in 62 % (grade 4: 4 cases), 28, and 27 % of patients, respectively. Acute radiation esophagitis of grades 3 and 4 was seen in 12 (10 %) and 0 patients, respectively. No other non-hematological severe toxicities (≥ Grade 3) like pain, pneumonia, dyspnea, nausea, and/or fatigue were seen in an acute/sub-acute phase.

### Late toxicities

In the ENI arm, in the late phase of 100 patients who achieved CR after CCRT, severe side effects (≥ grade 3) of the lung, heart, and esophagus were seen in 7, 5, and 2 patients, respectively (Table 2). No late side effect involving the skin or spinal cord was seen.

In the IFRT arm, each of grade 3 of recurrent nerve paralysis, grade 4 of laryngeal nerve dysfunction, or grade 4 of radiation pneumonitis was seen in 3 patients, respectively. Grade 3 of esophageal stenosis without recurrence of esophageal cancer was seen in two patients. There were three (4 %) treatment-related deaths, including sepsis in one patient (at 2.4 months after starting CRT), pneumonitis in one patient (at 2.0 months), and myelosuppression in one patient (at 2.5 months).

## Discussion

The aim of this study was to study the differences in outcomes between ENI and IFRT for esophageal cancer patients treated with definitive CRT. In the RTOG 85–01 trial [1], ENI was delivered at 30 Gy from the supra-clavicular fossae to the esophago-gastric junction, followed by cone down of 20 Gy to the primary tumor with 5 cm proximal and distal margins. On the other hand, in the INT0123 trial [2], ENI was omitted to improve the tolerance to treatment. In our institution, ENI had been used because the results of most surgical series in Japan have indicated a survival benefit of prophylactic 3-field LN dissection for SqCC in the thoracic esophagus [9]. To our knowledge, at present there are no other studies comparing ENI with IFRT in patients with esophageal cancer.

There are many reports about the results with evidence supporting a similar approach as this trial in non-small cell lung cancer [22–25]. They concluded that nodal failure rates in clinically uninvolved nodal stations were not increased with IFRT when compared to ENI and that IFRT significantly decreased esophageal toxicity in non-small cell carcinoma.

Cis-diammine-glycolatoplatinum (NDP) is a platinum derivative that was developed with the aim of reducing renal toxicity while maintaining the effectiveness of CDDP [26]. In a clinical study, combination chemotherapy using NDP and 5-FU has been reported to be a safe and effective method for treating advanced esophageal cancer [27]. Based on these facts, when the patient’s renal or cardiac function was poor, CDDP was replaced with NDP since 2000 [28].

According to previous randomized trials of esophageal cancer treated with CCRT, 2-year OS was 31–40 %, 3-year OS was 21–32 %, and the MST was 13.0–19.3 months [2, 29–31]. In the current study, 3-year OS was 35 % in ENI arm and 52 % in IFRT arm and the MST was 21 months in ENI arm and 39 months in IFRT arm. These results are comparable to the previous reports of randomized trials which used ENI.

The incidence of local/regional failure and the persistence of disease in the CRT arm of RTOG 85–01 [1] which used ENI, was lower than in the standard dose arm of INT0123 [2], which omitted ENI (46 % vs. 55 %). However, the MST and the 2-year OS rates were similar in both groups (14.1 months, 36 % vs. 18.1 months, 40 %), although the dose escalation trial by Minsky *et al.* [2] was influenced by premature deaths in the high-dose arm before reaching the high dose. In the current study, results of OS and PFS in IFRT arm were better than in the ENI arm. This may be because the number of cases given ≥ 2 cycles of chemotherapy was higher in the IFRT arm (104/119 cases) that in the ENI arm (92/120 cases). In fact, 3-year OS was significantly worse with only one cycle of chemotherapy (18 %) than with 2–4 cycles (42–54 %). Although at least two cycles of chemotherapy were tried to be administered in all cases, some patients in the ENI group were not able to be given even only one cycle due to the deterioration of performance status. The loco-regional recurrence outside the IFRT field in the IFRT arm did not increase, as compared to the ENI arm.

Since the radiation field was large in the cranio-caudal direction and included many thoracic vertebrae in the radiation field, the possibility was considered for myelo-suppression occurring more severely during treatment by chemotherapy in the ENI arm (grades 3–4 leukopenia and thrombocytopenia were 73 and 36 %, respectively) than in the IFRT arm (62 and 27 %). Thus, there may be fewer patients for a total cycle of chemotherapy in the ENI arm. There are some reports [32–34] indicating that the incidence of radiation esophagitis depends on the esophageal volume irradiated with a higher radiation dose. In the current study, grade 3–4 acute and sub-acute radiation-induced esophagitis was seen in 25 % of the ENI arm and in 10 % of the IFRT arm.

The limitations of this retrospective review are well recognized. The findings in this study were likely biased with significant differences in patient distributions of T stage, M1 stage, and performance status. Besides, the inhomogeneous use of PET in defining viable lymph node metastasis would alter the radiotherapy design. In Table 1, there are more T1-2 patients in the ENI arm, whereas there are more T4 patients in the IFRT arm. More patients with M1 disease were in the ENI arm. These may affect the treatment outcome data. Moreover, there were the higher amounts of censored events in the IFRT arm due to the shorter follow-up. Since the current 7th AJCC staging does not recognize M1a disease and the N classification is independent of tumor location, some stage IV patients included in this trial would be stage III or less by the 7th AJCC presumably.

Although the results of the INT 0123 study confirmed that higher radiation dose could not improve survival, the same irradiation dose with IFRT could decrease damage to normal tissues due to less normal tissues undergoing irradiation exposure than with ENI. This could encourage the use of IFRT, increasing its prevalence in CCRT. Ultimately, even a survival benefit may ensue. In summary, our results suggested that IFRT did not increase the risk of initially uninvolved or isolated nodal failures and loco-regional failure, although a substantial selection bias could not be excluded.

## Conclusions

The results of this study indicated that IFRT did not increase locoregional failure compared to ENI. It is thus suggested that further studies using IFRT are warranted.

## Abbreviation

CCRT: Concurrent chemoradiotherapy
3D-CRT: Three-dimensional conformal radiotherapy
SqCC: Squamous cell carcinoma
ENI: Elective nodal irradiation
LN: Lymph node
PTV: Planning target volume
IFRT: Involved-field radiotherapy
GTV: Gross tumor volume
CT: Computed tomography
FDG-PET: Fluorodeoxyglucose-positron emission tomography
CTV: Clinical target volume
AJCC: American Joint Committee on cancer
ICRU: International commisson on radiation units and measurements
OARs: Organs at risk
SUVmax: Standardized uptake value on the highest image pixel in the tumor regions
5-FU: 5-fluorouracil
NDP: Nedaplatin
CEA: Carcino-embryonic antigen
SCC: Squamous cell carcinoma-related antigen
CYFRA: Cytokeratin 19 fragment
ENF: Elective nodal failure
IFNF: Involved-field nodal failure
LPFS: Local progression-free survival
CTCAE: National cancer institute common toxicity criteria
MST: Median survival time
CI: Confidence interval
OS: Overall survival
